# Using the Spark Plug as a Sensor for Analyzing the State of the Combustion System

**DOI:** 10.3390/s25134198

**Published:** 2025-07-05

**Authors:** Matej Kučera, Miroslav Gutten, Daniel Korenčiak, Jozef Kúdelčík

**Affiliations:** 1 Department of Mechatronics and Electronics, FEEIT, University of Zilina, Univerzitná 1, 01001 Zilina, Slovakia; matej.kucera@uniza.sk (M.K.); miroslav.gutten@uniza.sk (M.G.); 2 Department of Physics, FEEIT, University of Zilina, Univerzitná 1, 01001 Zilina, Slovakia; jozef.kudelcik@uniza.sk

**Keywords:** spark plug waveform, fault model-based diagnosis ignition systems, spark plug as sensor

## Abstract

This article presents a method that uses a spark plug as a sensor to monitor an internal combustion engine. In addition, the voltage sensors measured the high voltage at the spark plugs using a non-contact method. Monitoring can now be performed in a simple way in real time, along with data processing. This method can be effectively used for the monitoring of all cylinders in an internal combustion engine as well as supplementing other measurement methods to optimize engine maintenance and enable correct diagnostic decisions to be made. Experimental analysis focused on the effect of the spark plug gap on the arc duration, flashover voltage, and high-voltage waveforms. It was found that with an increasing gap, the arc duration is shortened, and the breakdown voltage increases linearly, indicating wear of the spark gap. With increasing temperature, the breakdown voltage value decreased. Non-contact measurements at different frequencies showed a relationship between the magnitude of the electric field and the spark plug gap.

## 1. Introduction

Ignition and injection system diagnostics in internal combustion engines are performed by several methods, both during engine operation and out of operation. Stationary methods on a test rig allow for accurate damage assessment but are problematic due to the need to move the vehicle to a service center. Another diagnostic approach is model-based analysis, which determines the correct or incorrect operation of the measured system. Model-based diagnostics are very popular. In current solutions, model-based diagnostics could be performed automatically due to the implementation of algorithms used to evaluate the similarity between the datasets from the model and the measured data [1,2,3].

The reliability of data obtained from automotive sensors is currently still insufficient, as it cannot precisely detect a malfunction in the engine parts of a car. A comprehensive analysis of measured voltage waveforms of spark plugs is a unique way to understand what is happening in the combustion chamber, and the spark plug in its principle can fulfill the function of a sensor. By analyzing the history of recorded waveforms on the spark plug, we were able to determine whether it was an electrical malfunction in the ignition system, degradation of the spark plug, or an engine malfunction [4,5].

**Figure 1:**
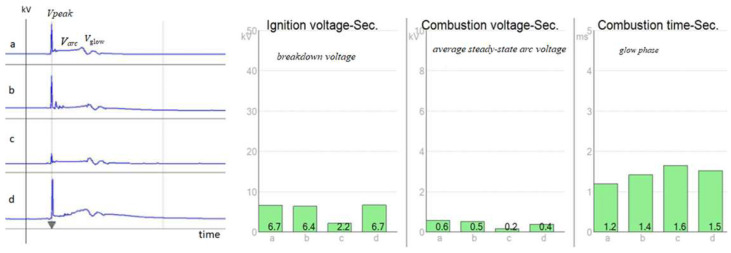
Secondary ignition analysis with a BOSCH FSA 720 module on a four-cylinder, four-stroke engine with new spark plugs.

The standard diagnostic measurement procedure on the secondary side of the ignition module of a four-stroke, four-cylinder MPI engine is presented in Figure 1. These parameters were measured by a BOSCH FSA 720 module on four cylinders. The results show that there is a clear deviation, and it is very difficult to determine what is causing the anomaly. It can be caused by a malfunction of the ignition module, degradation of the spark plugs, failure of the injector, pressure, or another reason. The implemented on-board diagnostic algorithms do not allow the service technician to diagnose the current condition without a history of records [6]. For this reason, historical records or often-used appropriate diagnostic methods or simulations are necessary.

Model analysis serves as a fundamental diagnostic approach for evaluating system operations. Recent advances allow for automated model-based diagnostics using algorithms that evaluate the similarity between model datasets and actual measurements [7,8,9]. Developing a mechanical model that accurately represents real-world processes is essential, as demonstrated in the analysis of injection systems by Hung et al. [10]. In addition, direct monitoring techniques using analysis of the secondary spark shape of spark plugs represent a significant diagnostic advance. The proposed devices for analyzing fault signals in ignition and injection systems can detect misfires during the combustion cycle and control re-ignition [10,11]. Simulations using a physical model of a spark plug have described the effect of the electric field between the electrodes under atmospheric pressure conditions [12]. While real spark discharges occur under high pressure, it has been found that the simulation results regarding the electric distribution and partial capacitance closely resemble real scenarios [12,13].

By analyzing key factors with advanced mathematical methods for comparing signal shape similarity signals with the implementation of degradation factors, we can use the spark plug (as a sensor) to more deeply analyze the processes in the engine combustion chamber and in its ignition system. Therefore, a physical model of the ignition system was created that would simulate the process state of the given system in real time, making it possible to analyze various fault conditions. Signal analysis by high-voltage probes was supplemented by non-contact diagnostics using analysis of the electromagnetic, thermal imaging, or acoustic field of the ignition system.

## 2. Theoretical Analysis of the Spark Plug as the Sensor of Internal Combustion Engine Processes

The ignition system consists primarily of a high-voltage module (ignition transformer), electronic components with power transistors, and protective elements together with a spark plug. The system is managed by signals generated by the control unit, which are sent to the power module. These signals activate the transistors, allowing the current to flow through the primary winding of the high-voltage transformer. When the transistor is turned off, energy transfers from the input to the output winding of a high-voltage transformer [14]. Modeling the spark plug during the ignition transformer discharge phase can be challenging, so it is recommended to first conduct a theoretical analysis of the ignition system under real operating conditions.

### 2.1. Physical Analysis of Spark Plug Breakdown Voltage

From the physical analysis of the breakdown voltage on a spark plug, it is evident that its value is a function of the pressure and the length of the gap. For determination of the breakdown voltage, Paschen’s law is used:

(1) $$V_{b}=\frac{Bpd}{\ln\left(Apd\right)-\ln\left(\ln\left(1+\frac{1}{\gamma }\right)\right)},$$

where

*p*—gas pressure,

*d*—electrode gap distance,

*A*, *B*—gas-specific constants,

*γ*—secondary electron emission coefficient [15].

Paschen’s law very accurately explains the influence of pressure and gap size on the ignition system. Initially, with an increasing gap, the ignition voltage decreases to a point of minimum voltage. Subsequently, with a further increase in the spark plug gap (0.1–1.1 mm), the voltage required to create an arc begins to increase approximately linearly. At atmospheric pressure and using plugs with a gap of 0.6–1.2 mm, good breakdown voltages (around 10–20 kV) can be achieved. However, if the spark plug is used as a sensor, temperature compensation must be implemented [16,17]. For a known value of the power dissipation, the temperature in the spark gap can be estimated using the relationship between the dissipated power and the heat required to increase the gas temperature.

### 2.2. Electrical Analysis for the Design of an Induction Ignition System Model

**Figure 2:**
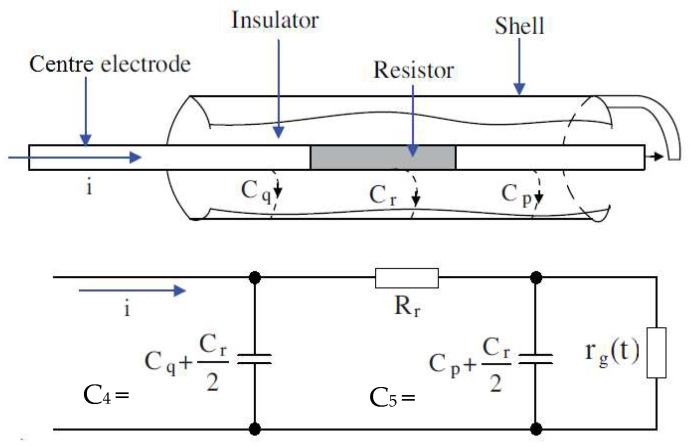
Spark plug and spark plug circuit model (according to [14], with modification [6]).

**Figure 3:**
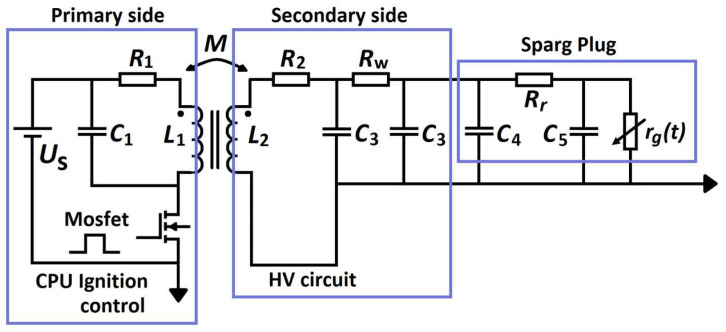
Circuit diagram of the induction ignition system (according to [11] with modification). *R*_1_, *R*_2_, and *L*_1_, *L*_2_ are the resistance and inductance of the primary and secondary windings of the transformer; *M* is the mutual inductance coefficient between the primary and secondary windings; *C*_3_ = C_r_/2 is the parasitic capacitance between the high-voltage conductor and the sheath; and *R*_w_ is the resistance of the high-voltage conductor.

In a spark plug analysis, the spark plug pulse is the main factor. This pulse is generated at the creation of an electrical breakdown in the spark plug, which is part of the ignition system. To create the mathematical description of a given process, it is necessary to correctly create an electrical model consisting of the parameters of the corresponding spark plug (Figure 2) and the parameters of the overall ignition system Figure 3 [18].

In Figure 2, *r*_g_ (t) is the spark plug air-gap resistance, which changes with time due to aging; *R*_r_ is the resistance of the conductive part; and *C_q_*, *C_p_*, and *C_r_*/2 are the parallel capacitances between the shell and the center electrode. From a comparison of Figure 2 and Figure 3, the next equation is produced [18]:

(2) $$C_{4}=C_{q}+\frac{C_{r}}{2},\;C_{5}=C_{p}+\frac{C_{r}}{2},\;C_{3}=\frac{C_{r}}{2},$$

Due to changes in parameters and the operational activity of the ignition system, the circuit has a dynamic nature and changes over time [19].

Figure 3 shows the circuit diagram of the electric induction ignition system used to compile the circuit equations of induction ignition. The ignition system itself consists of the following:

- CPU ignition control/engine control unit—primary side,
- HV circuit for generating high voltage/induction coils—secondary side,
- Spark plug, whose parameters degrade over time and are affected by mixture richness and pressure.

The ignition system circuit model in Figure 3 is shown with the following simplifications: (1) parasitic capacitances between the ignition system modules are neglected and are finally added to the entire system; (2) there is a simplified model of the ignition coil circuit, which is explained in [18]. From Figure 3, it is possible to solve the analysis of the ignition system by solving the system of equations (in a shortened version only for two states of the control block).

(3) $$U_{s}-i_{1}R_{1}-L_{1}\frac{di_{1}}{dt}+M\frac{di_{2}}{dt}=0$$

(4) $$L_{2}\frac{di_{2}}{dt}-M\frac{di_{1}}{dt}+i_{2}R_{2}+i_{3}R_{w}+u_{Sparg\;Plug}=0$$

(5) $$u_{c3}=L_{2}\frac{di_{2}}{dt}-M\frac{di_{1}}{dt}+i_{2}R_{2}$$

From the equivalent ignition circuit in Figure 3, the change from one energy state to another is primarily due to the change in energy *W*(*t*) of the electromagnetic circuit. This electromagnetic energy uses the electric field energy caused by the circuit capacitance *W_ek_*(*t*) and the magnetic field energy caused by the circuit inductance *W_mk_*.

(6) $$W\left(t\right)=\overset{n_{1}}{\underset{k=1}{\sum }}W_{ek}\left(t\right)+\overset{n_{2}}{\underset{k=1}{\sum }}W_{mk}$$

where *n*_1_ is the number of capacitors and *n*_2_ is the number of inductors.

Diagnostic systems work by evaluating the measured signals in certain modes and comparing them with the desired ones. Here, Equations (3)–(5) and others mathematically contained in Figure 3 were used.

## 3. Test Equipment

**Figure 4:**
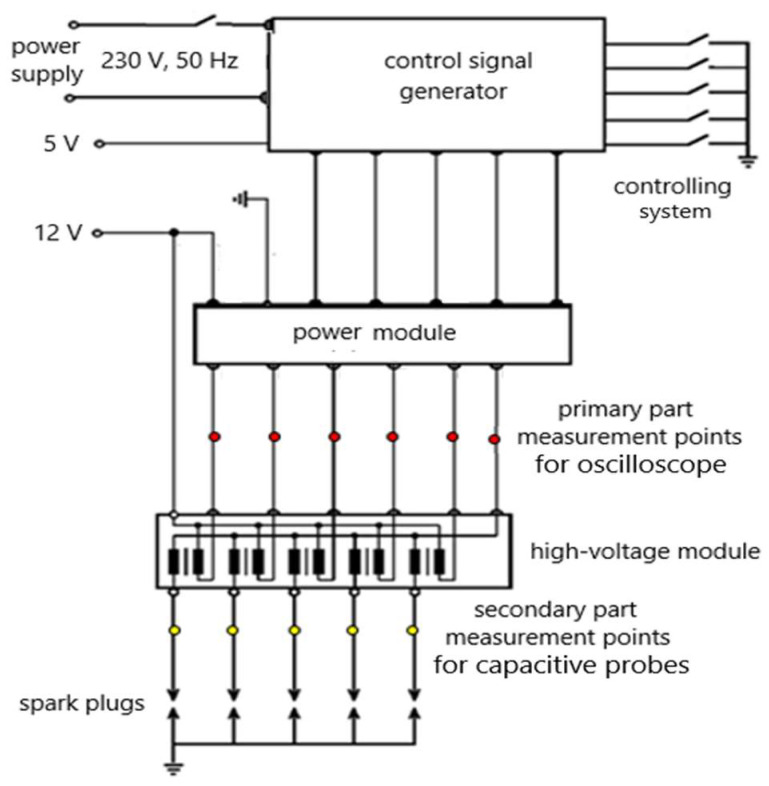
Ignition system test equipment wiring diagram.

Figure 4 shows the wiring diagram of the test equipment, which includes a control signal generator, power module, high-voltage module, and spark plugs. The diagram also includes measurement points for the direct connection of an oscilloscope (red and blue) and a capacitive probe (yellow).

The signal generator replaces the engine control unit in a simplified form. It is a programmed 8-bit microprocessor, DC9S08QE (AliExpress, China). Using the buttons, it is possible to activate the generator and change the time intervals during which energy is accumulated on the primary side of the ignition coils. The signal generator allows for the generation of the time t_on_ in the range from 0 to 9 ms and the time t_off_ in the interval from 10 to 500 ms with a step of 1 ms. Generation is performed for five channels. The display unit shows the times, the generator activation status, and the expected speed of the four-stroke engine. The generator produces a 5-volt voltage level. The output circuits contain optocouplers for galvanic isolation. For built-in online diagnostics, a voltage measurement test cycle was created for single timing intervals using measured numerical data. Since the sample obtained from each cylinder of the engine cycle is non-repeatable, four samples per cylinder are required. A pico-oscilloscope, 100 kHz, which is mainly used for measurements in cars, was used to capture the data.

A 12 V powered power module converts small 5 V voltage waveforms into power signals so that the spark plug is not oversaturated by means of safety elements. The high-voltage module is a block containing high-voltage ignition coils. More precisely, these are voltage transformers, the primary side of which has a resistance of 1.5 Ω and the secondary side of 5 to 10 kΩ. The primary sides of the coils are connected to a 12 V power supply, and the connection to the ground (vehicle chassis) is made using transistors.

For reliable analysis of the real operation of the ignition system and other engine parts subordinate to it (injection, optimization of the air–fuel mixture, operation of sensors, etc.), it is advisable to perform correctly determined diagnostic measurements, consisting of online, offline, or contactless systems. Thanks to the measured parameters, it is possible to conduct an in-depth inspection of several elements of the ignition engine, which allows the spark plug to be used as a sensor. A physical model was designed and implemented, thanks to which it is subsequently possible to obtain the necessary measured signals. The device allows for the creation of fault conditions and monitoring of changes in output characteristics.

## 4. Experimental Analysis

### 4.1. High-Voltage Spark Plug Waveforms

**Figure 5:**
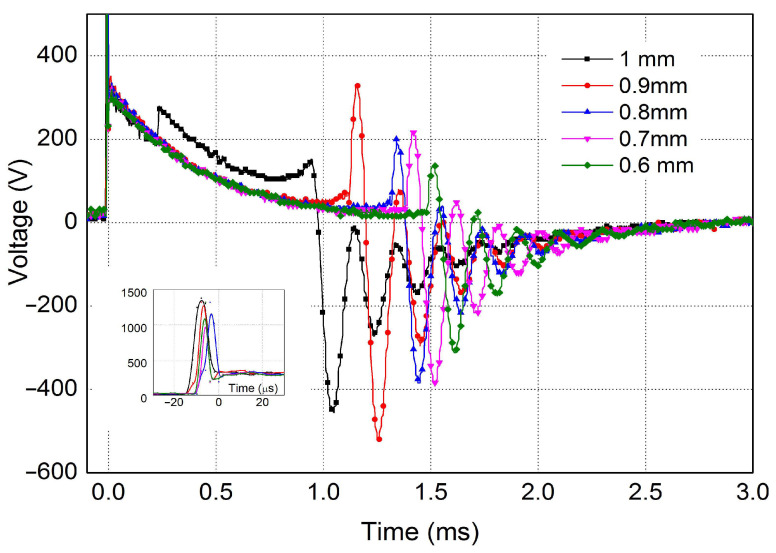
Analysis of the effect of spark plug gap on arc duration time using test equipment.

**Figure 6:**
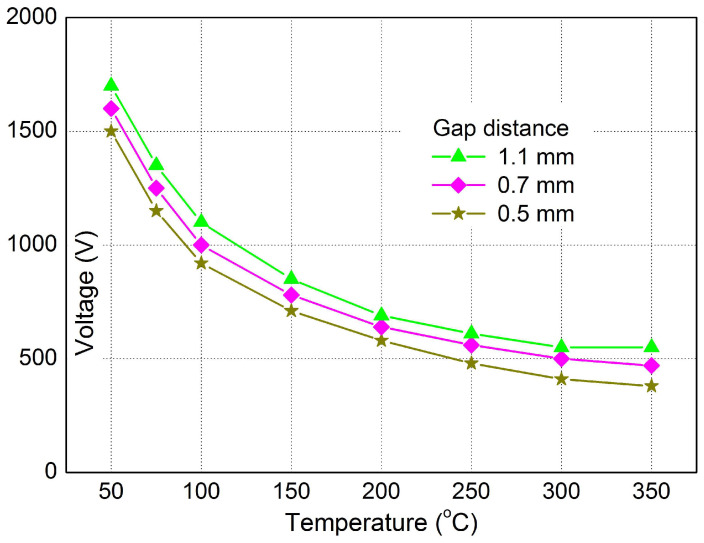
The temperature dependence of the breakdown voltage for different gap distances on the spark plug at constant pressure.

Spark plug replacement intervals for the latest types of ignition systems have been extended from 20,000 km to 120,000 km. Its improper operation can lead to the failure of other components in various systems. As the spark plug wears out, the gap between the electrodes (spark plug separation) increases. High-voltage curve measurements were performed on a test rig (Figure 5) to determine how the arc duration increases with increasing electrode gap. The voltage curve on new spark plugs of the same type with different electrode distances is shown in Figure 6. Considering the simulation in Figure 4, the measured curves were recorded only from time t_3_, and the oscilloscope triggering was set to the increase in ignition voltage.

Figure 5 shows the arc duration curves of a spark plug at different gap distances. The measured curves showed that the spark plug gap had a significant effect on the arc duration. As the distance increased, the arc duration also decreased. The difference between the minimum and maximum measured distances was as much as 0.55 ms.

A linear increase in breakdown voltage was observed with increasing plug electrode distance (Table 1). The measured values of the breakdown voltage in air also correspond to the voltage determined using the Paschen curve at the atmospheric pressure (according to Equation (1)). The observed increase in voltage therefore also corresponds to the wear of the spark gap. The higher the voltage, the faster the spark plug electrodes wear out. The decrease in pulse duration is also associated with an increase in the spark plug gap. A higher spike can indicate a wider gap, lean mixture, or high resistance; a lower spike may indicate a shorted plug or fouled plug.

The temperature dependence of the breakdown voltage for different gap distances on the spark plug at atmospheric pressure is shown in Figure 6. The temperature change in the spark plug placed on the test device was provided by a gas burner. In Figure 6, an exponential decrease in the breakdown voltage with temperature for three different distances can be seen, and from a value of 300 °C, an almost constant trend can be observed.

### 4.2. Non-Contact Analysis of the Spark Plug System

**Figure 7:**
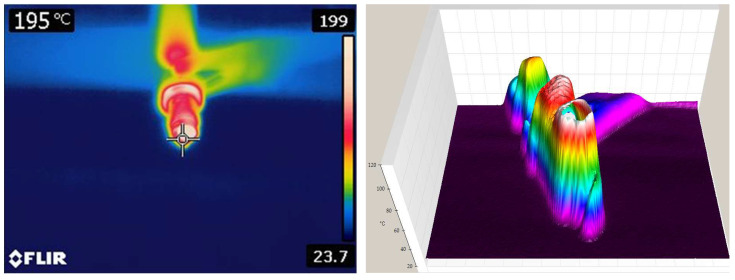
Two- and three-dimensional thermovision analysis of spark plug.

The temperature was measured by non-contact thermal field analysis using FLIR and FLUKE thermal imaging cameras (Figure 7). Further analysis created a 3D temperature model of the spark plug. Thermal imaging is a valuable tool for diagnosing engine problems by inspecting spark plugs. Using a thermal camera, we can detect temperature changes that indicate specific engine problems.

Thermal imaging can detect the following faults: the detection of misfires: a misfiring cylinder often shows a lower temperature in the exhaust manifold near that cylinder; air–fuel identification: with a lean mixture, the detected cylinder shows higher temperatures due to excess air; diagnosis of ignition problems: a faulty ignition coil or spark plug wire can be detected by uneven heat distribution; and assessment of engine load and timing: incorrect ignition timing causes uneven heating in the cylinders.

### 4.3. The Electric Field near the Spark Plug System

Currently, the so-called non-contact identification of the aging of the structural parts of the devices or their insulation using various correctly identified experimental measurement techniques (e.g., electromagnetic, thermographic, or acoustic field analysis) is advantageous for the diagnosis of electrical equipment issues during operation. The spark plug creates an electric arc, where a high current passing through the electric arc also generates a strong electromagnetic field, the detection of which can also provide information about the nature of the observed process. UHF signals of the electro-magnetic field can be measured using various antennas [20,21].

The above-mentioned measurement procedure uses a measurement technique in the range of very high frequencies measuring 100 kHz to 200 MHz. It was therefore not an electric field measurement directly related to spectral analysis of the waveform or FFT analysis, but rather the use of unwanted EMC interference on the electronic circuit of the car during ignition.

Non-contact measurements were performed on the testing system (Figure 5) too. The apparatus, a LUTRON EMF-819 (Micronics, Slovakia) 3-axis electromagnetic field meter with triaxial probes in the frequency range from 100 kHz to 200 MHz, was used to analyze the electromagnetic field. The measurement was performed at 0.5 m near the spark plug operation at various gap settings from 0.2 to 1.2 mm.

**Figure 8:**
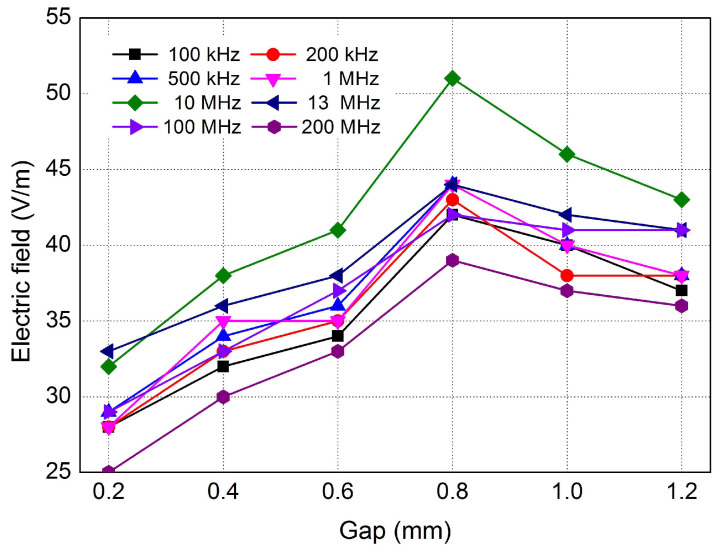
Relation of the magnitude of electric field and spark plug gap for various values of frequencies.

**Figure 9:**
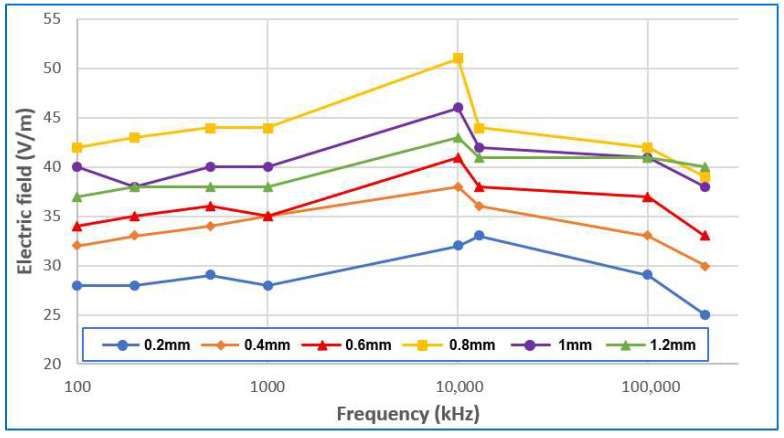
The dependence of the measured electric field magnitude in V/m on range frequency for various spark plug gaps.

The dependence of the measured electric field magnitude in V/m on the gap of the spark plug for various range frequencies is shown in Figure 8. According to Figure 8, the magnitude of the electric field gradually increases up to a spark plug gap of 0.8 mm and then decreases slightly at larger gaps, which is best observed during measurement at a frequency of 10 MHz. The most unstable frequencies during the measurement (larger ripple during gap rise) were reported at the lowest values, i.e., at 100 and 200 kHz, and the lowest electric field values were reported at the measured frequency of 200 MHz. For all spark plug sparks, the most beneficial frequency for the measurement was 10 MHz, where the highest possible electric field values were identified. For analyzing the magnitude of the electric field, the frequency listed is the best choice for the reliability of the given measurement. According to Figure 9, for all spark plug breaks, the most beneficial frequency for the measurement was 10 MHz, where the highest possible electric field values were identified. For analyzing the magnitude of the electric field, the frequency listed is the best choice for the reliability of the given measurement.

At larger gap distances of greater than 0.8 mm, the electromagnetic field interference was attenuated due to changes in the behavior of the electric discharge, and in particular, the instability of the arc. At larger electrode distances, the ignition coil must generate a higher voltage to overcome the increased dielectric resistance of the air. This leads to the discharge (arc) becoming more unstable and shorter. Instead of consistent burnout, discontinuous, intermittent or weaker discharges occur, which produce a less intense electromagnetic field.

The above measurement results have also been confirmed by some studies. According to the authors of [22,23,24], the high frequencies of the electric field in the megahertz (MHz) range that appear when measuring the electric field during the ignition of a spark plug are caused by the sharp and rapid course of the spark discharge, which is characterized by a short duration and a high voltage gradient. Sharp changes in voltage and current in the ignition system generate strong electromagnetic interference—so-called radio waves, which can extend into the radio spectrum (AM/FM ranges) and therefore contain frequency components in MHz. Therefore, ignition often causes interference in radio equipment if it is not well shielded.

According to the authors of [22], the analysis confirms that typical spark plug ignition produces high-frequency spikes, with significant energy present in the bands below 30 MHz. These emissions are caused by rapid voltage transients at the inrush and a break in the ignition coils, creating harmonics and sharp spikes.

## 5. Discussion

Thanks to the set of proposed measurements on the physical model, it is possible to create reference operational and error measurements, based on which we can use them to identify the operation of the real engine system. Using the test rig, it is possible to perform an experimental analysis of the simulated aging of automotive components.

In total, demonstration examples of faults were simulated on both the physical and real models and selected as a set of single and combined faults. Several types of single faults are shown in Table 2, and several types of combined faults were created, which had a significant impact on the shape of the high-voltage waveform on the spark plug. However, there may be a problem where the combined fault patterns are not caused by a random combination of single faults but by some real combinations (e.g., it is not possible to have a large spark plug break and a small spark plug break at the same time). Moreover, experimental data show that the combined fault pattern of the ignition system is formed by a combination of, at the most, three single faults. In addition to these limitations, it is not possible to capture ignition patterns due to engine stalling.

The breakdown voltage of the spark plug demonstrated a positive correlation with the electrode gap distance (Figure 5), aligning with the principles of the Paschen curve. Conversely, the arc duration exhibited an inverse relationship with the electrode gap, as detailed in Table 1. These fundamental relationships serve as indicators of spark plug wear, where an increased gap (due to wear) corresponds to higher breakdown voltages and shorter arc durations.

Our analysis of high-voltage waveforms reveals several critical parameters for evaluating ignition performance: the pulse length, the duration of the spark burning, the magnitude of the step voltage amplitude, and the amplitude of the burn. As is illustrated in Figure 5, our masking technique is sensitive not only to amplitude errors in the measured signal but also to deviations in the shape of the waveform and timing relative to the established time base.

**Figure 10:**
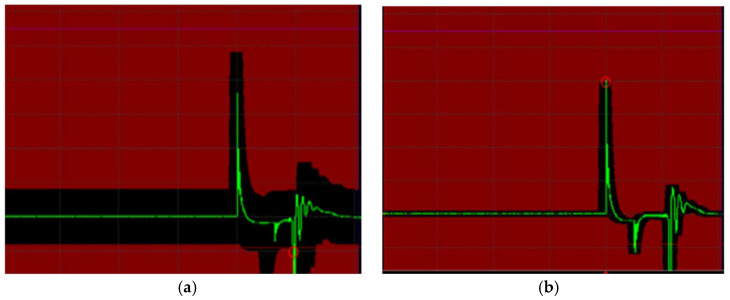
Measurement of tolerance band mask for partial failure detection thresholds of (**a**) 10% and (**b**) 2%.

By establishing a reference combustion waveform, we can define tolerance bands to identify both partial failures and degradation indicative of the need for component replacement. As is depicted in Figure 10, the first tolerance band can serve as a threshold for detecting partial failures, while a second, wider band can indicate complete ignition system inactivity during the arc burning interval.

The mask, generated based on the current waveform with a defined percentage tolerance, provides a visual means of assessing waveform conformity. When a measured waveform encroaches upon the mask’s boundaries (represented as the red zone in the graph), an overlap is flagged with a red circle in the comparison window (Figure 10b). This indicates that the waveform deviates beyond the set tolerance (e.g., 2% in our example). The software can classify consecutive waveforms as “good” (within the mask) or “bad” (touching the mask), enabling real-time monitoring and long-term sensor data logging.

The evaluation of residual evaluator performance extends beyond simple failure detection, offering insights into the gradual degradation of component properties. Monitoring the arc duration, in conjunction with the described waveform analysis using tolerance masks, presents a robust approach for both identifying existing malfunctions and predicting the progression of spark plug degradation. Future efforts will focus on leveraging machine learning techniques to analyze these waveform characteristics and tolerance band violations, aiming to achieve accurate fault classification and reliable component prognosis. This will involve determining the optimal algorithms and feature sets derived from the waveform parameters and mask comparisons to maximize diagnostic and prognostic accuracy.

## 6. Conclusions

A significant contribution of this research was the demonstration of the possibilities of using the electrical analysis of voltages of the spark plug in order to obtain information about its wear and combustion quality.

The result of the theoretical part of this study was the proposal of a new model of the ignition system with the possibility of connecting additional components and creating a new simulation environment in the LabVIEW system. By combining them, it is possible to increase the reliability of the system for detecting a fault and possibly creating a prognosis for components in an engine.

In the experimental part of this study, diagnostic procedures were used for the analysis of the ignition system using time and non-contact measurements. The measurements clearly show an increase in the arc duration with an increasing electrode gap. A decrease in the breakdown voltage with increasing temperature is also clear from the measurements. The method of measuring the electromagnetic field using the UHF signals confirmed the measurement was best at 10 MHz.

## Figures and Tables

**Table 1:** Characteristic parameters of breakdown time voltage at various gas distances.

Gap mm	Breakdown Voltage V	Time ms
0.5	1420	1.60
0.6	1500	1.50
0.7	1540	1.40
0.8	1600	1.30
0.9	1680	1.18
1.0	1740	1.05
1.1	1820	0.95

**Table 2:** Types of uniform errors resulting from the ignition system.

Case No.	Symptom or Possible Failure
1	Problems in the injection system
2	Misfire due to rich or lean fuel mixture
3	A large gap between the electrodes of the spark plug due to its aging
4	Mechanical failure in the engine (engine knocking)
5	Dirt-clogged ignition or injection system

## Data Availability

The original contributions presented in this study are included in the article. Further inquiries can be directed to the corresponding author.
